# Evaluation of a proprietary software application for motion monitoring during stereotactic paraspinal treatment

**DOI:** 10.1002/acm2.13594

**Published:** 2022-03-26

**Authors:** Qiyong Fan, Hai Pham, Pengpeng Zhang, Xiang Li, Tianfang Li

**Affiliations:** ^1^ Department of Medical Physics Memorial Sloan Kettering Cancer Center New York New York USA

**Keywords:** IMR, KV imaging, markerless spine tracking, paraspinal SBRT

## Abstract

**Purpose:**

Stereotactic paraspinal treatment has become increasingly popular due to its favorable clinical outcome. An often‐overlooked factor that compromises the effectiveness of such treatment is the patients’ involuntary intrafractional motion. This work introduces and validates a proprietary software application that quantifies such motion for accurate patient monitoring during treatment.

**Methods:**

The software uses a separate full‐trajectory cone‐beam computed tomography (CBCT) after daily patient setup to establish reference projections. Once treatment starts, the software grabs the intrafraction motion review (IMR) image acquired by TrueBeam via the Varian iTools Capture software and compares it against the corresponding reference projection to instantly determine the 2D shifts of the vertebrae being monitored using the classical downhill simplex optimization method. To evaluate its performance, an anthropomorphic phantom was shifted 0, 0.6, 1.2, 1.8, 2.4, 3.0, and 5 mm in three orthogonal directions, immediately after the full‐trajectory CBCT but prior to treatment. Depending on the scenario of shift, a nine‐field fixed gantry intensity‐modulated radiation therapy (IMRT) plan and/or a four partial‐posterior‐arcs volume‐modulated radiation therapy (VMAT) plan were delivered. For the IMRT plan, three IMR images were acquired sequentially every 200 monitor units (MU) at each treatment angle. For the VMAT plan, one IMR image was acquired every 15° of each arc. For each IMR image, the software‐reported 2D shift was compared with the ground truth. Certain tests were repeated with 1°, 2°, and 3° of rotation, pitch, and roll, respectively. Some of these tests were also repeated independently on separate days.

**Results:**

Based on the group of tests that involved only the IMRT delivery, the maximum standard deviation of the software‐reported shifts for each set of three IMR images was 0.16 mm, with 95th percentile at 0.02 mm. For translational shift, the maximum registration error was 0.44 mm, with 95th percentile at 0.23 mm. Left unaccounted for, rotation and pitch degraded the registration accuracy mainly in the longitudinal direction, while roll degraded it mainly in the lateral direction. The degradation of registration accuracy is positively related to the degree of rotation, pitch, and roll. The maximum registration errors under 3° rotation, pitch, and roll were 2.97, 1.44, 2.72 mm, respectively. Based on the group of tests that compared IMRT delivery with VMAT delivery, the registration errors slightly increased as magnitude of shifts increased; however, they were well under the 0.5‐mm threshold. No significant differences in registration errors were observed between IMRT and VMAT deliveries. In addition, the variation in registration errors among different days was limited for both IMRT and VMAT deliveries.

**Conclusions:**

Our proprietary software has high repeatability, both intrafractionally and interfractionally, and high accuracy in registering IMR images with the reference projections for motion monitoring, regardless of the magnitude of shifts or treatment delivery technique. Rotation, pitch, and roll degrade registration accuracy and need to be accounted for in the future work.

## 1 INTRODUCTION

Stereotactic body radiation therapy (SBRT) has been increasingly popular in recent years due to the advancement of technology in all aspects of radiation therapy that enabled high‐precision treatment. Among all the disease sites being treated with SBRT, the lesions of spine and paraspinal regions have garnered more and more interest from radiation oncologists. This is largely due to the fact that a plethora of studies by different institutions have generally reported improved survival and relatively low toxicity rate with paraspinal SBRT when compared to standard fractionation treatment.^1^, ^2^, ^3^, ^4^, ^5^, ^6^, ^7^, ^8^, ^9^, ^10^

The critical technical issues of stereotactic paraspinal treatment lie in the intent of delivering a large amount of dose, up to 24 Gy per fraction,^11^ millimeters from the spinal cord. Consequently, the clinical workflow of stereotactic paraspinal treatment must ensure this ablative dose is delivered with high precision. Excellent progresses have been made accordingly in the major steps of paraspinal SBRT: from simulation that provides accurate delineation of spinal cord to treatment planning that generates steep dose gradient.^12^, ^13^ However, an often‐overlooked factor that may compromise the otherwise effective clinical workflow is the patient's involuntary intrafractional motion, especially in situations where patients are immobilized only by evacuated cushion‐type device.^14^

Due to the high‐precision nature of the paraspinal SBRT, the checking of setup images by physicians and/or physicists is necessary. In addition, the treatment plan usually has a large amount of monitor units (MU), given the high fractional dose and complex modulation needed. As a result, the delivery of paraspinal SBRT often lasts tens of minutes, despite the use of flattening filter‐free (FFF) beams. This timescale of delivery gives enough room for patient movement to happen. In fact, Agazaryan et al. found that the vertebrae could move up to 3 mm in 5 min, while patients were instructed to maintain still.^15^ Considering the steep dose gradient, close proximity of spinal cord, and the ablative amount of dose, a movement of a few millimeters could have made significant adverse dosimetric impact that limits the therapeutic ratio of the treatment.^16^, ^17^ Therefore, it is imperative to have intrafractional patient movement monitoring during the entire radiation delivery.

A few systems that are commercially available to address the above issue include (a) Xsight spine tracking on the CyberKnife platform (Accuray Inc, Sunnyvale, CA)^18^, ^19^; (b) ExacTrac x‐ray 6D system (BrainLAB AG, Feldkirchen, Germany),^20^, ^21^ both of which employ an orthogonal pair of kilovoltage (KV) x‐ray imaging systems mounted in fixed positions. However, for conventional linear accelerator (Linac) equipped with a single KV onboard imager (OBI) and megavoltage (MV) electronic portal imaging device, the above systems are either not available or entail significant cost financially and inconvenience clinically. The most common solution provided by conventional Linac is an imaging technique called intrafraction motion review (IMR) image, enabled by the OBI and provided in the modern Linac such as Varian TrueBeam (Varian Medical System, Palo Alto, CA). With this technique, therapists collect IMR images throughout the treatment to decide if the vertebra being monitored moves during treatment. This is achieved by visual comparison of IMR image against the digitally reconstructed radiograph (DRR) with the help of a selected region of interest (ROI). The drawback of this technique is two‐fold: (a) visual comparison can be subjective and hence cannot be effectively enforced clinically; (b) the daily difference between planning computed tomography (CT) and setup cone‐beam CT (CBCT) cannot be fully accounted for in the above comparison, even after a reasonable registration, thereby introducing errors in evaluating the intrafraction motion.

This work introduces and evaluates a proprietary application that was developed at our institution and can be integrated with the Varian TrueBeam system seamlessly. The initial version of the software, called SequenceReg, was developed in our institution to monitor prostate and lung treatments.^22^, ^23^ In this work, we are adding the software's capability to monitor the patient movement during paraspinal SBRT under a separate module (referred to as “the software” onward for simplicity). With certain modification, the software can be made versatile to work with any conventional Linac system that has a KV imager. It can be used clinically to solve the aforementioned issues of the IMR technique by (a) using the daily CBCT directly as the reference for motion evaluation, and (b) providing quantitative motion magnitude along with clinical tolerance for therapist intervention. This application can help therapists definitively determine when to beam off and re‐do setup imaging. This work also aims to establish a standard procedure of using the software and determine the baseline criteria for motion tracking accuracy and uncertainty. For the performance evaluation, extensive studies have been carried out for both intensity‐modulated radiation therapy (IMRT) and volume‐modulated radiation therapy (VMAT) delivery techniques, including a total of 86 tests combining different shifts and rotations to simulate the intrafractional motion.

## 2 MATERIALS AND METHODS

### 2.1 The software interface

**FIGURE 1:**
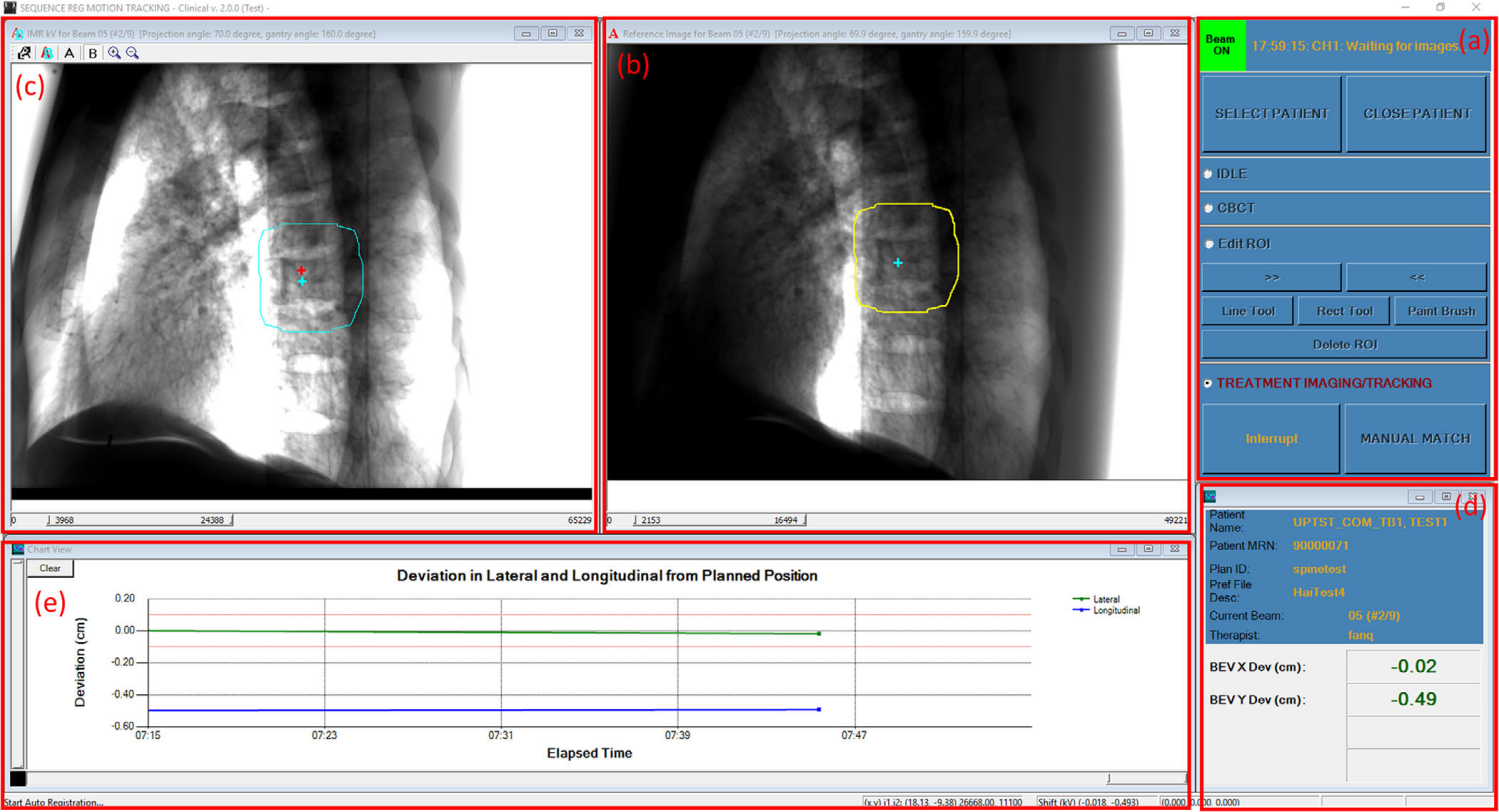
The interface of the proprietary software consisting of five major panels: (a) workflow panel; (b) reference image panel; (c) intrafraction motion review (IMR) image panel; (d) patient/plan info and tracking results panel; and (e) graphic results panel

Written in a combination of C++ and C# language, the software is installed on a dedicated workstation located close to the Linac console. The software utilizes the Varian iTools Capture software to continuously grab the KV images from the frame grabber. The interface of the software is shown in Figure 1, which consists of five major panels: (a) *Workflow panel*: this panel is mainly used to guide the workflow of use. The top bar indicates the status of both the beam (i.e., beam on or beam off) and the software (e.g., waiting for CBCT projections, waiting for images, etc.). Beneath this bar, users can SELECT PATIENT to be treated or CLOSE PATIENT when treatment is finished. The software offers four operational modes sequentially that tracks the workflow of use: (i) the IDLE mode puts the software in idle so users can perform other procedures that are irrelevant to the software; (ii) the CBCT mode is used to collect reference CBCT projections for tracking purpose; (iii) the EDIT ROI mode gives users an opportunity to check the ROI on every relevant reference projection and make necessary edits using the provided contour editing tools such as paint brush; and finally (iv) the TREATMENT IMAGING/TRACKING mode (for simplicity, referred to as TRACKING mode onward) allows users to track the vertebrae motion as treatment progresses. A manual‐match function is provided in this mode if user intervention is needed. (b) *Reference image panel*: this panel displays the reference projection applicable to the current treatment angle. Window and level are automatically set to properly display the images and can be adjusted. The cyan‐colored crosshair indicates the projected isocenter on the reference projection, while the yellow‐colored contour indicates the ROI for tracking. (c) *IMR image panel*: this panel displays the IMR image acquired by OBI in real time in a similar way as the reference image panel. The two crosshairs shown in this panel indicate the projected isocenter on the acquired IMR image (cyan) and the reference projection (red), respectively. Therefore, the distance between these two crosshairs is a measure of patient movement from reference position. (d) *Patient/plan information and tracking results panel*: this segment shows the tracking result, that is, two‐dimensional (2D) shift of the selected ROI in both lateral and longitudinal directions, along with the identifying information about the patient and the plan. (e) *Graphic results panel*: this depicts the tracking results versus time graphically.

### 2.2 Clinical workflow of using the software

**FIGURE 2:**
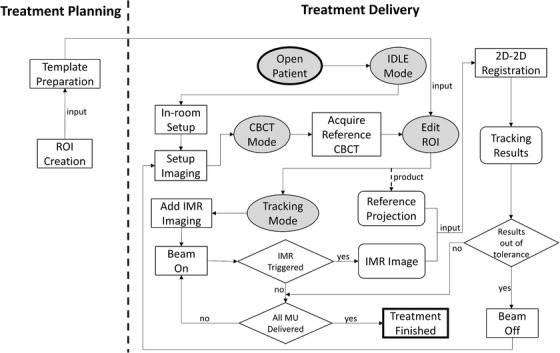
Clinical workflow of using the software. There are two parts of this workflow: treatment planning and treatment delivery. During the treatment planning, planners need to create the ROI for tracking. At treatment delivery, the use of the software is designed to be seamlessly integrated with the routine of the therapists. All the operations relevant to the software that therapists need to perform are highlighted by the shading oval shape: (1) open patient, (2) enter IDLE mode, (3) enter CBCT mode, (4) enter EDIT ROI mode, and (5) enter TRACKING mode. All these operations can be achieved via one or two mouse clicks. Other operations are the same as the therapists would normally perform, that is, setup the patient in‐room and via imaging, add intrafraction motion review (IMR) imaging on console, beam on and beam off. The software will process any qualified IMR images and provide tracking results by performing a 2D/2D registration between the reference projection and IMR image. The therapists are instructed to beam off and re‐do setup imaging if the tracking results are out of the established clinical tolerance

The overall clinical workflow of using this software application is depicted in Figure 2, which includes two components: treatment planning and treatment delivery. During treatment planning, the planner needs to prepare an ROI for tracking. This is achieved by first contouring the vertebrae of interest on the planning CT and then expanding it with a 2‐cm margin in all directions. Using a proprietary Eclipse plugin, this ROI is automatically projected onto the imaging plane at 150 cm source to imager distance (SID) and saved as ROI template files, which later are used to display ROI on both the reference projection and IMR image, as shown in panels (b) and (c) of Figure 1.

At the beginning of the treatment delivery, therapists first select the patient on the software and then go through the four operational modes sequentially, as described in Section 2.1. Specifically, therapists first put the software in IDLE mode so that they can attach IMR imaging templates to the treatment fields, set up the patient in‐room, and acquire CBCT to align the patient as they normally do. After finishing setting up the patient, therapists put the software in CBCT mode and initiate another CBCT scan on Linac console to acquire the reference projections for tracking. Note that this reference CBCT scan needs to be acquired in the full trajectory mode so that the scan contains sufficient reference projections applicable to all treatment angles. After the reference scan is completed, the ROI templates generated during the planning process are automatically overlaid on the corresponding reference projections and displayed in the panel (b) of Figure 1. Therapists or physicists may enter the EDIT ROI mode to check and edit the ROI projections at all relevant beam angles, if needed. The ROI editing principles include (a) to make sure ROI covers mostly the bony anatomy; (2) to move the ROI away from any moving soft tissues such as diaphragm; (3) the ROI needs to be as small as possible but covers at least one to two vertebral bodies. Any ROI changes will be saved automatically in the ROI template files and passed on to the future fractions. Finally, therapists enter the TRACKING mode and click on the "START" button to initiate the tracking so that the software is waiting for IMR image to be grabbed and tracked.

After entering the TRACKING mode, therapists can treat as they normally do and monitor the tracking results on the software (i.e., panel d of Figure 1). Therapists continue to deliver the treatment plan if the tracking results are within the established clinical tolerance. When the tracking results are out of tolerance, therapists are instructed to beam off, re‐do setup imaging, re‐acquire the reference CBCT, and restart the above treatment cycle. Once beam on, the Linac acquires IMR images based on the specified triggering MU or gantry angle. These IMR images are grabbed by the software in real time and are paired with the corresponding reference CBCT projection whose angle is both closest to and within a 0.75° interval of that of the IMR image. The software then performs a 2D/2D registration and almost instantly, the tracking results are displayed on the software for therapists to monitor.

### 2.3 Image acquisition and motion‐tracking algorithm

A Varian TrueBeam Linac (version 2.7) with a Perfect Pitch 6‐DoF couch was used to acquire the IMR images and deliver the test treatment plans. Full trajectory and full fan scan protocol was used for the reference CBCT scans, and each scan had about 895 projection images, resulting in an angle interval of about 0.4°. This ensures the angle difference between the IMR image and the reference projection is less than 0.2°, which is assumed to cause negligible uncertainty to the registration results.

A Varian 4030CB OBI was used to acquire IMR images when triggered. All images were acquired at 150 cm SID. The active imaging area of the OBI was 39.7 × 29.8 cm^2^ with a 1024 × 768 pixels array. The x‐ray tube model was Varian GS 1542. The IMR imaging setting on TrueBeam console was “thorax arms up with small size” for all test cases, which yielded imaging parameters of 100 kV and 5 mAs. Conventional corrections to the IMR images were applied by TrueBeam based on the routine monthly PVA calibration, which included dark field and flood field calibrations, profile correction, and bad pixel corrections.

The images were automatically grabbed from OBI with the Varian iTools Capture software at a rate of about 14.8 fps. After a CBCT projection or an IMR image was read in by the software, the tracking ROI saved in the template files was overlaid on the image based on the DICOM coordinates. The average intensity of pixels inside the tracking ROI was then calculated, the value of which decided how images were processed depending on the image type. For CBCT projections, the grabbed frame was saved as references when the average intensity is larger than 40. Such minimal filtering will reject blank frames but ensures all required reference projections were saved. For IMR images, only when the beam was on, the average pixel intensity was between certain thresholds, and the intensity was more than twice that of the previous frame, this frame was added to the queue for the 2D/2D registration to avoid unqualified images. In our study, the lower and upper thresholds we used were 1500 and 200 000, respectively. Note that these values are configurable. All other frames that were grabbed will be rejected for processing. Compared to the minimal filtering for CBCT projections, the stronger filtering for IMR images will ensure only one IMR frame per KV beam is processed for registration.

In this work, the motion‐tracking algorithm is based on the principle of template‐based matching in our prior work.^24^, ^25^ Conventionally, template‐based matching is primarily focused on the tracking of radio‐opaque objects such as implanted fiducial markers and is often based on planning CT images (i.e., DRR). As there are usually no implanted markers involved with paraspinal SBRT treatments, our algorithm instead tracks the vertebrae of interest as defined by the tracking ROI. In addition, our algorithm uses CBCT projections right before the treatment to establish the tracking template or reference as DRR‐based template has three disadvantages: (a) DRR has different level of image intensity than that of the IMR image; (b) DRR does not share the same systematic error of the KV imaging system as that of IMR image (e.g., KV imaging isocenter concordance with machine isocenter, or KV imaging isocenter walk); and (c) DRR‐represented planning position may still deviate from the daily treatment position, even after a reasonable CBCT registration. The inaccuracy of registration resulted from the above disadvantages may be small; however, it might still be significant in the context of submillimeter‐level motion tracking. Therefore, to eliminate such potential inaccuracy, we acquired a separate CBCT scan after patient setup and just before radiation delivery to specifically establish a reference (noted as $\mathrm{Pro}j_{r}$) for real‐time IMR image (noted as $\mathrm{Pro}j_{i}$) to compare to.

The motion was quantified by the 2D image shift, in beam's eye view at the isocenter plane, between the IMR image and the reference image. This 2D shift was determined by finding a solution that maximizes an objective function of normalized cross‐correlation inside the tracking ROIs, as follows:

(1) $$\mathrm{Obj}\left(x,y\right)=\frac{\mathrm{Corr}\left[T_{2D\left(x,y\right)}\left(\mathrm{Pro}j_{r}\right),\;\;\mathrm{Pro}j_{i}\right]}{\left\|\mathrm{Pro}j_{r}\right\|\cdot \left\|\mathrm{Pro}j_{i}\right\|}\;,$$

where Obj(x,y) is the objective function, (x,y) represents the relative 2D shift between the IMR image and reference CBCT projection, and $T_{2D(x,y)}$ is a 2D translation inside the beam's eye view. The objective function value ranges from 0 to 1, where a larger value indicates better match.

To search the solution, denoted as $\left(x_{\mathrm{opt}},\;y_{\mathrm{opt}}\right)$, for the above objective function, the classical 2D downhill simplex optimization method was used.^26^ In actual implementation, the sign of the objective function was flipped, and hence minimization was instead performed. The optimization started with an initial simplex located at $\left(x_{0},\;y_{0}\right)$, $\left(x_{0}+\Delta s,\;y_{0}\right)$, and $\left(x_{0},\;y_{0}+\Delta s\right)$, and

(2) $$\left(x_{0},\;y_{0}\right)=\left\{\begin{array}{c} \left(0,0\right)\;\;\;\;first\;IMR\;image\\ {\left(x_{\mathrm{opt}},\;y_{\mathrm{opt}}\right)}_{\mathrm{prev}}\;\;\;\;subsequent\;IMR\;images\; \end{array}\right.,$$

where ${\left(x_{\mathrm{opt}},\;y_{\mathrm{opt}}\right)}_{\mathrm{prev}}$ was the optimized 2D shift from previous IMR image.

Δs was the initial searching step size. In each iteration of the optimization, a series of operations, including reflection, expansion, contraction, and shrink, were performed based on the classical downhill simplex optimization algorithm.^26^ Optimization stopped when the following condition was met:

(3) $$2\frac{\left|\mathrm{Ob}j_{H}-\mathrm{Ob}j_{L}\right|}{\left|\mathrm{Ob}j_{H}\right|+\left|\mathrm{Ob}j_{L}\right|}<\sigma ,$$

where $\mathrm{Ob}j_{H}$ and $\mathrm{Ob}j_{L}$ represented the highest and the lowest objective function value within the same simplex, respectively, and σ represented the threshold tolerance for terminating optimization. The vertex location when the optimization stopped was selected as $\left(x_{\mathrm{opt}},\;y_{\mathrm{opt}}\right)$, which was reported as the 2D shift on the software. If the difference did not fall below *σ* after a maximum of *N* iterations, the optimization reinitialized at its current location with the initial step size Δs. This was referred to as a simplex rebuild. Our algorithm continued the optimization for *n* times of simplex rebuilds unless the aforementioned difference fell below *σ*. Note that only the pixel values inside the ROI were used for optimization.

For the tests in this work, Δs = 2 mm, *σ* = 10^−6^, *N* = 20, and *n* = 4. The capture range, defined as the maximum lateral and longitudinal distance from the current position the search algorithm can move, was set at 2 cm (also configurable). To prevent the algorithm from finding a solution outside the capture range, the objective function returned the worst normalization cross‐correlation value when evaluating the vertices located outside the capture range during the optimization. The latency between image capture and tracking results display was estimated to be less than 200 ms.

### 2.4 Performance evaluation

#### 2.4.1 Phantom and treatment plans

In this study, we used a thorax anthropomorphic phantom called “LUNGMAN” (Kyoto Kagaku Co., Japan) to test the performance of our software. The phantom contained synthetic bones made of epoxy resin and soft tissue made of polyurethane. The phantom size was 43 × 40 × 48 cm^3^ with chest girth of 94 cm, and the weight was approximately 18 kg.

**FIGURE 3:**
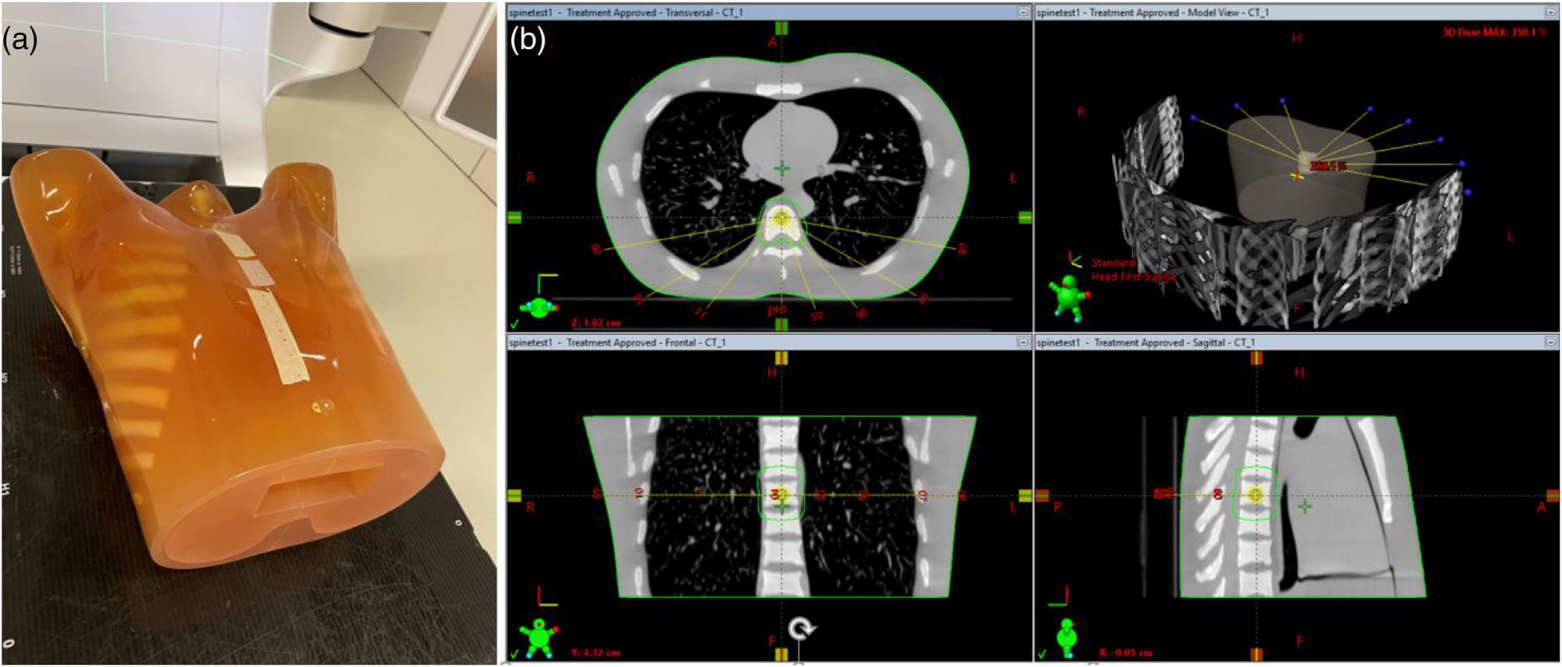
(a) Anthropomorphic phantom used in the study, and (b) testing plan consisting of nine posterior beams located at gantry angles 180°, 160°, 140°, 120°, 100°, 260°, 240°, 220°, and 200°, respectively

Treatment plans delivered using both IMRT and VMAT techniques were used to evaluate the software. For IMRT delivery, an automated approach to intensity‐modulated treatment planning, the expedited constrained hierarchical optimization (ECHO) engine, has been incorporated into the Eclipse treatment planning system and is used to generate clinical IMRT plans for spine SBRT treatments at our institution.^13^ Under this treatment planning technique, nine posterior 6‐MV FFF beams are used to treat the thoracic and lumbar spine lesions. The nine gantry angles are typically 180°, 160°, 140°, 120°, 100°, 260°, 240°, 220°, and 200°. In this study, we transferred a typical clinical ECHO plan to this phantom with a target at the T7 vertebra. As the aim of this study was to investigate the motion‐tracking accuracy of our algorithms, the modulation of the ECHO‐generated treatment plan was irrelevant and hence each beam was reset to an open field of 2 × 2 cm^2^ with 410 MU to facilitate evaluation. This field size was chosen to represent the average aperture opening of the original plan, and hence the influence on IMR image quality due to treatment beam scatter was similar.^27^ In addition, IMR imaging was set to be triggered every 200 MU, and hence three IMR images were acquired during the delivery of each beam. Figure 3 shows the anthropomorphic phantom used and the fixed gantry IMRT test plan setup.

For VMAT delivery, a clinical VMAT plan created using the Eclipse built‐in Photon Optimizer was transferred to the phantom with a similar plan setup. The VMAT plan consisted of four partial posterior arcs covering an angle range of [50 179] and [290 181] and was delivered using 6‐MV beams. IMR images were triggered every 15° of each arc, resulting in a total of 20 images per treatment.

#### 2.4.2 Test designs

The software performance to be evaluated includes the intrafractional and interfractional repeatability of the algorithm, the image registration error in different shift directions and under different amounts of shifts, how rotational shifts (i.e., pitch, roll, rotation) affect the tracking accuracy given the software cannot track rotational motion, and how treatment delivery technique (i.e., IMRT vs. VMAT) changes the tracking error. To effectively evaluate the above metrics, the tests have been grouped into two categories: IMRT‐only delivery, and IMRT versus VMAT delivery.

##### IMRT‐only delivery

To evaluate the intrafractional repeatability of the algorithm, the general motion‐tracking accuracy in different shift directions, and how tracking accuracy can be affected by rotational shifts, 50 IMRT‐plan‐based tests simulating different combinations of shift directions and rotations were performed. Five tests involved only 3D translational shift, which includes zero shift, 5‐mm superior–inferior shift, 5‐mm anterior–posterior shift, 5‐mm right–left shift, and 5‐mm shift in all three orthogonal directions. To evaluate how rotation, pitch, and roll affect the registration algorithm performance, the same series of five tests was repeated nine times separately with added 1°, 2°, and 3° of rotation, pitch, and roll, respectively. Except the type of shift applied, the test workflow was the same among all 50 tests, as depicted in Figure 2. Shift was introduced by shifting couch, and immediately following the reference CBCT acquisition but before beam delivery to introduce the difference between the reference projection and the IMR image during treatment.

##### IMRT versus VMAT delivery

To evaluate the interfractional repeatability of the algorithm and how the magnitude of shifts as well as the delivery technique (IMRT vs. VMAT) can affect the registration error, 12 additional tests with a 0, 0.6, 1.2, 1.8, 2.4, and 3.0 mm shift in all three orthogonal directions were performed under IMRT and VMAT delivery techniques, respectively. Besides the treatment plan, delivery technique, and the IMR image triggering method, everything else was the same including the test workflow as indicated in Figure 2. These 12 tests were repeated altogether on two other separate days, resulting in a total of 36 tests.

## 3 RESULTS

### 3.1 IMRT‐only delivery

#### 3.1.1 Intrafractional repeatability of the algorithm

**FIGURE 4:**
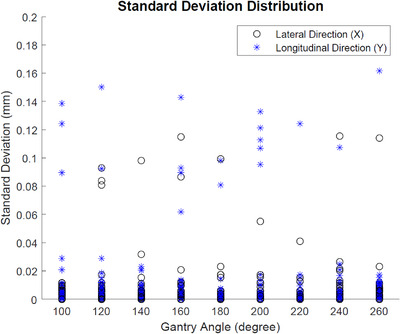
The standard deviation distribution as a function of the gantry angle

In order to evaluate how repeatable the tracking algorithm is intrafractionally, we designed the tests so that there were three IMR images triggered for every beam of each test. As there were three registration results per beam, we calculated a standard deviation for those three results. Ideally the standard deviation should be zero, as the phantom did not move between three images. The smaller the deviation, the more repeatable the tracking algorithm is. In this study, in total we had 900 data points due to a combination of 50 tests, nine IMRT beams, and two directions (i.e., lateral and longitudinal directions). We plotted these data points versus the gantry angles, as shown in Figure 4.

It can be observed that most of the data points are below 0.05 mm, with the 95th percentile at 0.02 mm. The maximum standard deviation is 0.16 mm, a value that is still very small. All these results indicate that the software has good repeatability intrafractionally.

**FIGURE 5:**
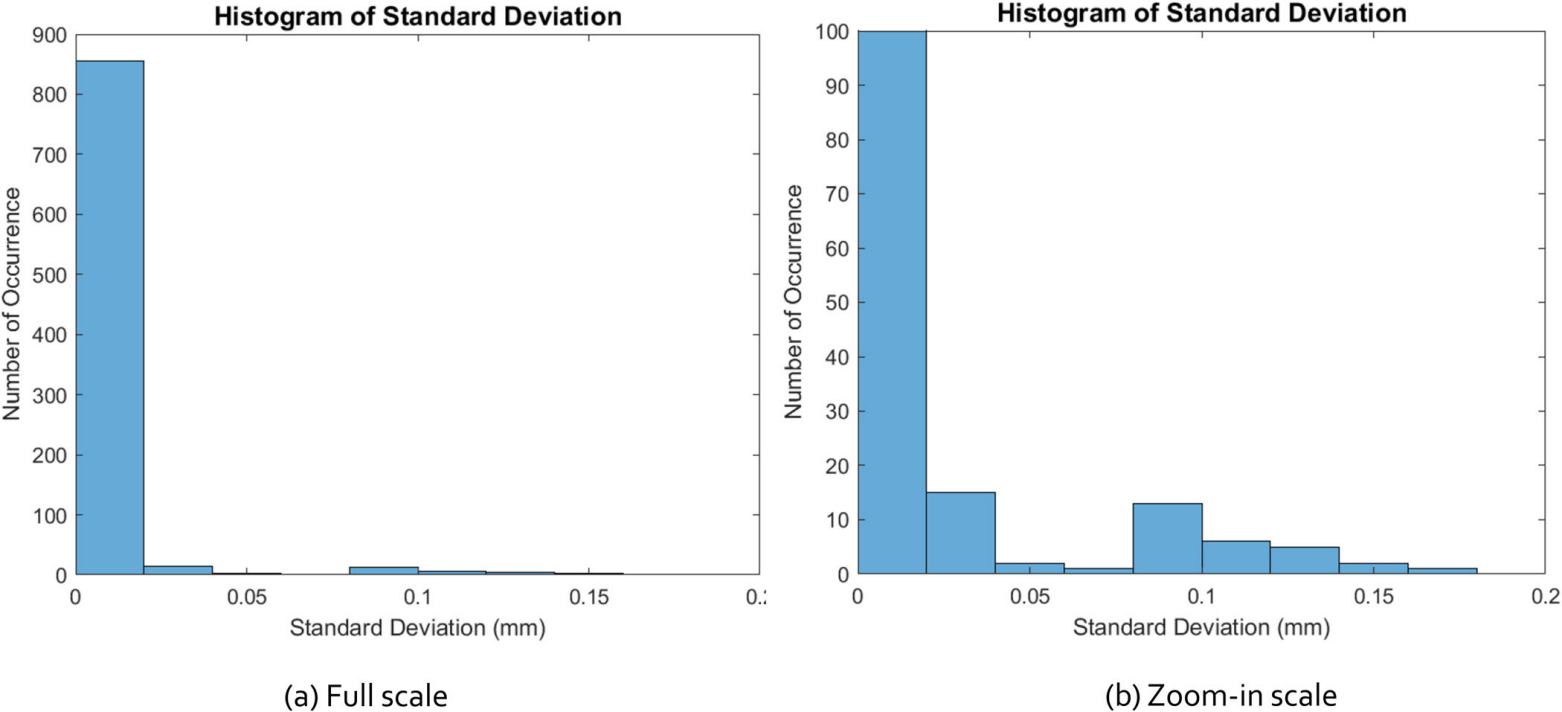
(a) Full scale, and (b) zoom‐in scale histograms of the standard deviation

We then plotted the histogram of these 900 data points of standard deviation, as shown in Figure 5. In the left, we have a full‐scale histogram where one can see that more clearly most data points fall below 0.05 mm, while on the right we have a zoom‐in scale histogram where it shows the data points that are above 0.05 mm are on the order of 10 counts or less (<1.1% of all occurrences).

**FIGURE 6:**
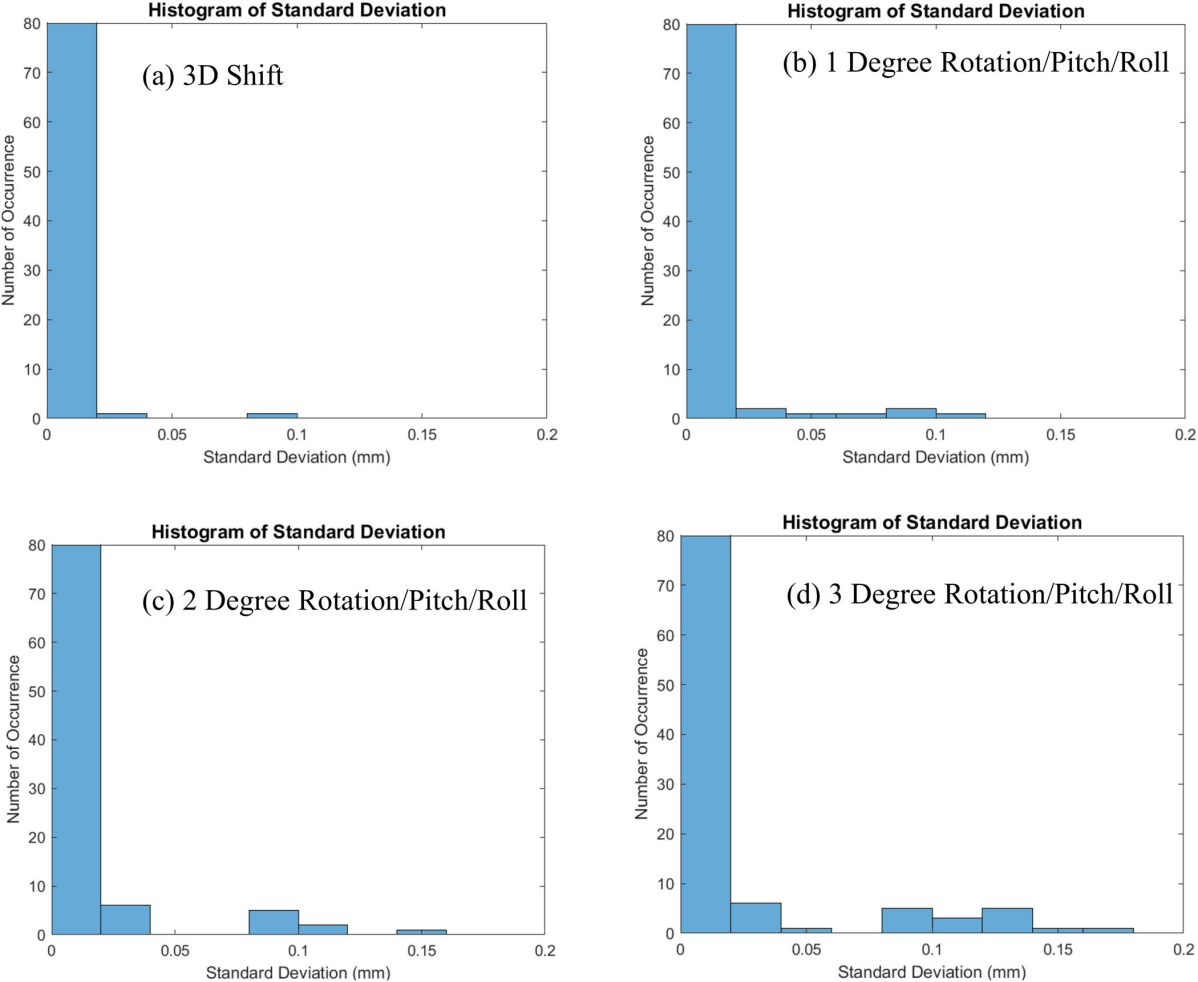
Histogram of standard deviation under (a) 3D shift only, (b) 1°, (c) 2°, and (d) 3° of rotation, pitch, and roll, respectively

Finally, we plotted the histogram of the standard deviation under different types of rotational shifts using the zoom‐in scale in Figure 6. As it is clearly shown, as more of the rotation, pitch, and roll exist, one can observe more counts of large standard deviation (i.e., larger than 0.05 mm). This is an indication that rotation, pitch, and roll can degrade the repeatability of the algorithm.

#### 3.1.2 Registration error distribution with only 3D translational shifts

**FIGURE 7:**
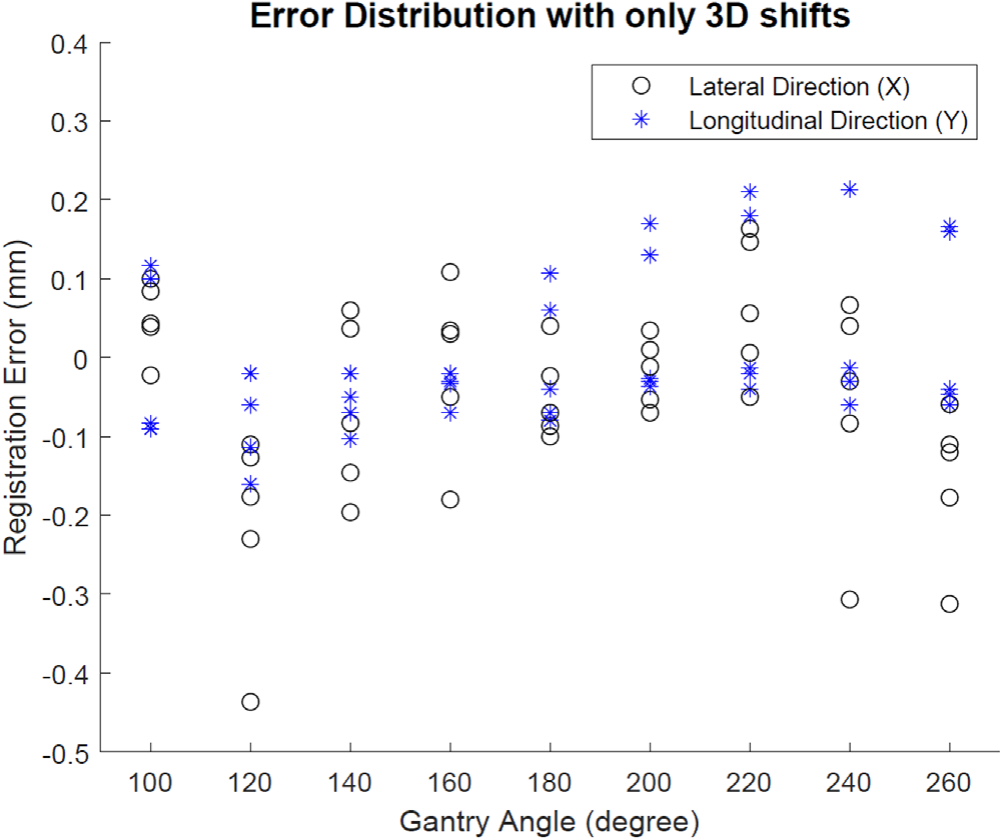
Error distribution with 3D shifts only versus gantry angle

The registration error was calculated as the difference between the software‐reported 2D shift and the expected shift (i.e., the ground truth). The results are summarized in Figure 7 and Table 1. It is shown that the maximum error was 0.44 mm, while most of the data points are within a ±0.20 mm range. In fact, the 95th percentile of the registration errors were 0.31, 0.21, and 0.23 mm for lateral, longitudinal, and both directions, respectively. These results indicate that the software achieved a submillimeter tracking accuracy with a phantom movement up to 5 mm in all directions, which represented an upper limit of typical motion magnitudes that can be encountered during paraspinal SBRT treatments.

**TABLE 1:** Maximum and 95th percentile tracking error with intensity‐modulated radiation therapy (IMRT)‐only 3D shifts (in absolute value)

	Lateral direction (*x*)	Longitudinal direction (*y*)	Both directions
Maximum (mm)	0.44	0.35	0.44
95th Percentile (mm)	0.31	0.21	0.23

#### 3.1.3 Registration error distribution with both 3D shift and rotation/pitch/roll

**FIGURE 8:**
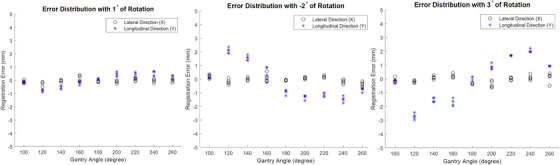
Error distribution with 3D shifts and 1°, 2°, and 3° of rotation

**FIGURE 9:**
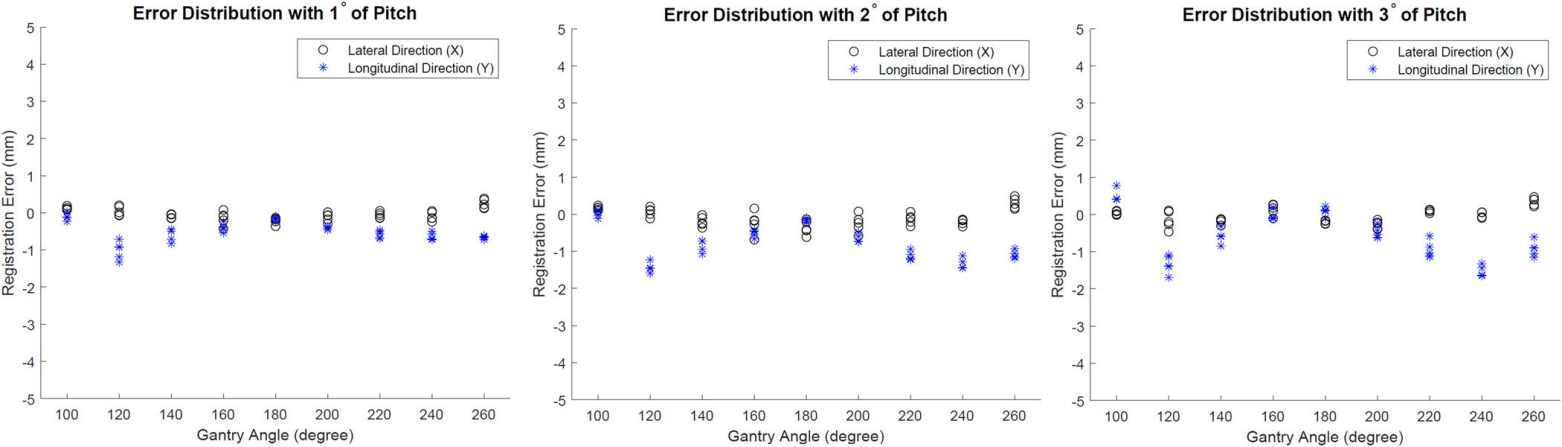
Error distribution with 3D shifts and 1°, 2°, and 3° of pitch

**FIGURE 10:**
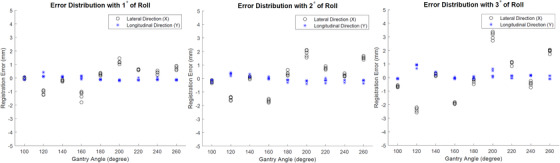
Error distribution with 3D shifts and 1°, 2°, and 3° of roll

In the series of tests with rotation, pitch, and roll, the registration error calculation was the same as above, that is, as if there were no rotation/pitch/roll applied, to evaluate how rotation/pitch/roll might affect the registration accuracy when they were unaccounted for. Results are shown in Figure 8 (rotation), Figure 9 (pitch), and Figure 10 (roll), respectively. It seems that these parameters can increase the registration error, although primarily in either lateral or longitudinal direction but not both, and additionally in certain consistent gantry angles but not all angles. In addition, pitch exerted least impact when compared to rotation and roll. While these results quantify the impact of rotation, pitch, and roll on the overall registration accuracy for the current set of test phantom and plan, it should be understood that such impact can depend on other factors such as the size and location of the ROI.

As shown in Figure 8, under 1° of rotation, the maximum error does not exceed 1 mm in both directions. This indicates the current software can tolerate certain degree of rotation. As rotation increases, the maximum error exacerbates, especially at angles 120° and 240°. In addition, rotation seems to only affect the registration accuracy in longitudinal direction but not the lateral direction. The pattern of influence is very consistent among different degrees of rotation. It is worth mentioning that during the test, we intentionally reversed the sign of rotation for 2° case to test how the pattern of influence might change. The results show that as sign of rotation flips, the pattern of influence is also reversed. This indicates that the influence by the rotation is anything but random, which allows possible systematic correction.

Figure 9 shows the degradation of registration accuracy by pitch. Similarly, pitch affects the registration accuracy mainly in longitudinal direction and the worst influence happens at 120° and 240° gantry angles. The maximum error caused by 3° of pitch does not exceed 2 mm.

The impact of registration accuracy caused by roll is depicted in Figure 10. Unlike rotation and pitch, roll mainly affects the registration accuracy in the lateral direction. The impact is mainly focused on gantry angles of 120°, 160°, 200°, and 260°. Even under 1° of roll, the maximum error is almost 2 mm, which indicates the registration of current software is least tolerant to the impact of roll.

It should be noted that regardless of the type of rotational shift, the registration error is less pronounced at gantry angle of 180° when compared to other gantry angles such as 120° or 200°. Besides the measurement uncertainty, this is most likely because when the tracking ROI is located at the isocenter, the 2D/2D registration is less prone to error when the imaging projection direction is either perpendicular or parallel to the rotational axis.

### 3.2 IMRT versus VMAT delivery

This group of tests aims to evaluate the interfractional repeatability of the software and how the registration accuracy might be affected under different magnitudes of shifts as well as by different delivery techniques (i.e., IMRT vs. VMAT). Table 2 summarizes the maximum and 95th percentile registration error on three separate days for a 0, 0.6, 1.2, 1.8, 2.4, and 3.0 mm shift in all three directions under IMRT and VMAT deliveries, respectively. It can be observed that (a) registration errors slightly increased as magnitude of shift increased; however, the registration errors (both maximum and 95th percentile error) were well under 0.5 mm regardless of the shift amount; (b) the registration errors did not show significant differences between IMRT and VMAT deliveries, which was as expected, as delivery technique exerts limited impact toward the IMR image acquisition and image registration; and (c) the variation in registration errors among different days was limited for both IMRT and VMAT deliveries, which indicated a good interfractional repeatability of the software.

**TABLE 2:** Maximum and 95th percentile tracking error with intensity‐modulated radiation therapy (IMRT) versus volume‐modulated radiation therapy (VMAT) deliveries (both directions, in absolute value)

	**IMRT delivery (mm)**						**VMAT delivery (mm)**					
**Shift cases (mm)**	**Day 1**		**Day 2**		**Day 3**		**Day 1**		**Day 2**		**Day 3**	
	**Max**.	**95th**	**Max**.	**95th**	**Max**.	**95th**	**Max**.	**95th**	**Max**.	**95th**	**Max**.	**95th**
0	0.14	0.13	0.19	0.17	0.16	0.15	0.21	0.14	0.17	0.11	0.23	0.16
0.6	0.18	0.18	0.21	0.20	0.23	0.23	0.32	0.21	0.27	0.19	0.27	0.20
1.2	0.19	0.19	0.23	0.23	0.19	0.19	0.30	0.27	0.33	0.28	0.28	0.23
1.8	0.33	0.33	0.31	0.30	0.28	0.28	0.36	0.30	0.33	0.26	0.39	0.33
2.4	0.35	0.35	0.33	0.33	0.30	0.29	0.39	0.31	0.38	0.33	0.34	0.30
3.0	0.39	0.37	0.34	0.32	0.37	0.35	0.42	0.34	0.40	0.30	0.38	0.32

**FIGURE 11:**
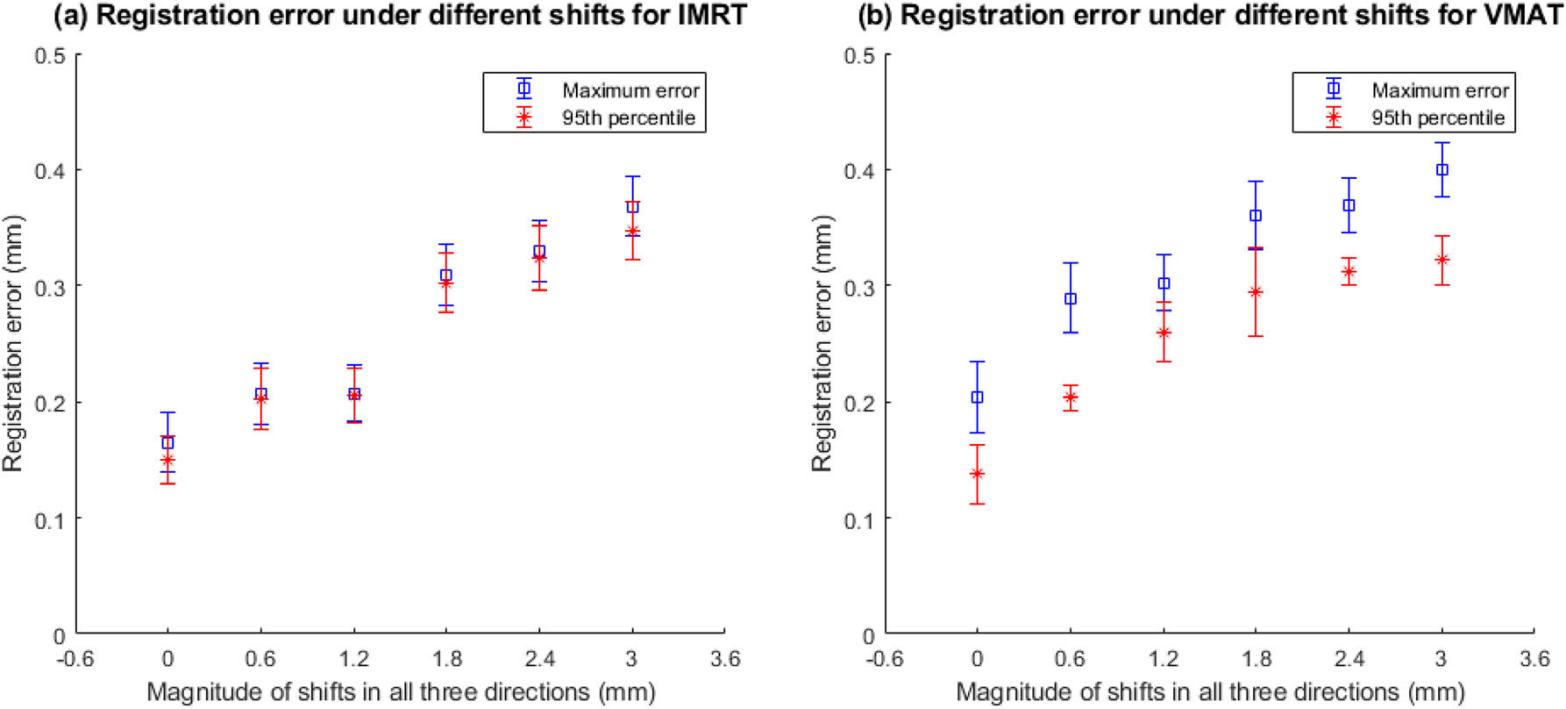
Registration error under different magnitudes of shifts in all three directions ranging from 0 to 3.0 mm in 0.6‐mm increment for (a) intensity‐modulated radiation therapy (IMRT) and (b) volume‐modulated radiation therapy (VMAT) deliveries. Depending on the amount of shift, standard deviation of each data point (or half of the error bar) ranges from 0.01 to 0.04 mm

The above results can be more clearly visualized in Figure 11, which plots the registration errors as a function of the magnitude of shifts in the format of two error bar graphs for IMRT and VMAT deliveries, respectively. Note that the mean and standard deviation (i.e., half of the error bar) of each data point were calculated out of the three independent tests on three separate days. It is worth mentioning that the difference between maximum and 95th percentile error was significantly lower for IMRT delivery than that for VMAT delivery. This was due to a much smaller number of data points in IMRT delivery, causing the 95th percentile of the data to be much closer to its maximum.

## 4 DISCUSSION

We have developed a software application to monitor intrafractional patient movement during paraspinal SBRT treatment. The design and implementation of the software stem from a close collaboration between the clinical physicists and software engineers, and match carefully with the intended clinical workflow of motion monitoring and management. To test the software, we designed and carried out a series of relevant experiments that simulated the actual clinical use, which revealed potential advantages and pitfalls of this application. These experiments may serve as standard tests for commissioning and/or routine QA of the system.

Despite stellar repeatability and great accuracy, the software comes with certain limitations that we are working on to address. The biggest limitation is that the software only provides 2D tracking and hence it cannot detect the motion in the imaging projection direction. The work to solve this issue is currently in progress and our approach is to incorporate the concurrent MV imaging enabled by treatment beams. The feasibility of this approach was demonstrated and recently published,^28^ and we are working to include it as part of the software. Regardless of this work in progress, current software still provides a useful tool for motion‐monitoring purpose. This is because compared to depicting the motion accurately in 3D, the more important question to answer clinically is whether the patient has moved during treatment. Our current software can provide an adequate answer. Furthermore, the testing procedure established in this work can be naturally adapted to MV imaging as well.

Another concern that one might raise is the need of a separate CBCT scan every fraction to establish reference for tracking, which increases the imaging dose significantly. It should be emphasized that this software application will be mainly used for paraspinal SBRT treatment, which usually has no more than three fractions. Therefore, the benefit of patient monitoring in this ablative dose setting outweigh the risk of elevated imaging dose. In addition, as the second CBCT can be used for verification, the KV setup imaging prior to the first CBCT can be skipped to compensate for the increased imaging dose. It is also our long‐term goal to eliminate the need of this second CBCT by improving the software to directly create reference projections out of the first CBCT.

People might also be concerned about the limited capture range of the software (currently 2 cm in both lateral and longitudinal direction). Indeed, due to the requirement of low latency, the search area for image registration cannot be unlimited; however, this should not limit the system to detect a motion larger than 2 cm, as the cross‐correlation will drop significantly for such motion. Also, the purpose of the software is to determine whether patient has a movement that is larger than the clinical tolerance, which is on the order of a few millimeters. When a big motion happens (e.g., >2 cm), although the software might not be able to register this motion with great accuracy, it will certainly alert the therapists to intervene, which serves the purpose of motion monitoring well.

In addition, it should be noted that all the phantom studies have been performed without immobilization devices. However, it is expected that the immobilization devices would cause very limited impact to the tracking accuracy as (a) they do not degrade the image quality in a meaningful way, (b) they remain stationary during the entire tracking process, and (c) the devices will not be included in the ROI for registration.

Finally, rotation, pitch, and roll currently cannot be accounted for as the determination of such parameters requires the information beyond what can be detected by a single OBI imager. The ultimate solution needs to make use of information that is concealed in the treatment beams^28^ as previously mentioned and it is a work in progress. Nevertheless, it should be noted that 3° of rotation, pitch, or roll is very significant motion, and it can translate to large 2D shifts as demonstrated by the phantom studies, by which RTTs can be alerted to stop the beam. For more realistic situation, such as 1° of rotation, pitch, and roll, the results show that our software can still track the spine motion with adequate accuracy.

## 5 CONCLUSION

Our work introduced a software tool for motion monitoring in stereotactic paraspinal treatment and described a procedure for testing the software. Through extensive tests, it is shown that the software has high repeatability, both interfractionally and intrafractionally, and high accuracy in detecting 3D translational shift, regardless of the magnitude of shifts or treatment delivery technique. Rotation, pitch, and roll will adversely affect the registration accuracy.

## CONFLICT OF INTERST

The authors declare they have no conflicts of interest.

## AUTHOR CONTRIBUTION

Qiyong Fan designed and performed the tests, analyzed the test results, and drafted the manuscript. Hai Pham and Pengpeng Zhang were responsible for the production of the software based on the feedback from all other co‐authors. Xiang Li provided guidance and feedback on the software development and testing procedure. Tianfang Li participated in test design and oversaw the entire project. All co‐authors participated in the manuscript editing.
